# Oral Treatment with d-RD2RD2 Impedes Early Disease Mechanisms in SOD1*G93A Transgenic Mice but Does Not Prolong Survival

**DOI:** 10.3390/biomedicines11040995

**Published:** 2023-03-23

**Authors:** Katharina Wintz, Julia Post, Karl-Josef Langen, Dieter Willbold, Antje Willuweit, Janine Kutzsche

**Affiliations:** 1 Institute of Biological Information Processing, Structural Biochemistry (IBI-7), Forschungszentrum Jülich GmbH, 52425 Jülich, Germany; k.wintz@fz-juelich.de (K.W.); j.post-schulz@web.de (J.P.); d.willbold@fz-juelich.de (D.W.); 2 Institute of Neuroscience and Medicine, Medical Imaging Physics (INM-4), Forschungszentrum Jülich GmbH, 52425 Jülich, Germany; k.j.langen@fz-juelich.de; 3 Department of Nuclear Medicine, RWTH Aachen University, 52062 Aachen, Germany; 4 Institut für Physikalische Biologie, Heinrich-Heine-Universität Düsseldorf, 40225 Düsseldorf, Germany

**Keywords:** amyotrophic lateral sclerosis, survival study, behavior, motor coordination, d-enantiomeric peptides, SOD1*G93A

## Abstract

Amyotrophic lateral sclerosis (ALS) is a neurodegenerative disease affecting upper and lower motor neurons, thus, progressing to complete muscle loss until the patient dies from respiratory arrest. The disease is not curable, and patients die approximately 2–5 years after diagnosis. Studying the underlying disease mechanisms to get access to new treatment options is, therefore, essential for patients’ benefit. However, so far, only three drugs that alleviate the symptoms have been approved by the U.S. Food and Drug Administration (FDA). A new drug candidate for the treatment of ALS is the all-d-enantiomeric peptide RD2RD2. In this study, we investigated the therapeutic effect of RD2RD2 in two setups. First, we analyzed disease progression and survival in 7 week-old B6.Cg-Tg(SOD1*G93A)1Gur/J mice. Second, we confirmed the result of the survival analysis in the B6SJL-Tg(SOD1*G93A)1Gur/J mouse line. Shortly before disease onset, the mice were treated daily with an oral dose of 50 mg/kg body weight. Treatment with RD2RD2 led to a delayed disease onset and reduced motor phenotype as shown using the SHIRPA test, the splay reflex test, and the pole test, but did not affect survival. In conclusion, RD2RD2 has the ability to delay the onset of symptoms.

## 1. Introduction

The d-enantiomeric peptide RD2RD2 belongs to a novel class of peptide drugs solely consisting of d-amino acid residues. In contrast to L-enantiomeric peptides, they possess beneficial properties such as high proteolytic stability and low immunogenicity [1]. RD2RD2 is the head-to-tail tandem version of the d-peptide RD2 [2] which successfully passed phase I clinical trials in humans for the treatment of Alzheimer’s disease (AD) [3]. RD2RD2 was also originally designed for the treatment of AD. In a proof-of-concept study in a transgenic AD mouse model, a prominent effect of RD2RD2 on inflammation was observed. Subsequently, it was tested in a mouse model of amyotrophic lateral sclerosis (ALS) [4,5], another neurodegenerative disease known to be strongly influenced by inflammatory processes [6].

ALS is a neurodegenerative disease with a progressive loss of upper (UMNs) and lower (LMNs) motor neurons in the cortex, brain stem, and spinal cord. Early symptoms are muscle weakness, wasting, and spasms, later leading to a complete loss of muscle function. Most patients die from respiratory arrest [7]. ALS can be distinguished by the type of onset (limb onset 65%, bulbar onset 30%, respiratory onset 5%) and by genetic predisposition (10% familial cases (fALS) vs. 90% sporadic cases (sALS)) [8]. To date, there are 18 genes classified as definitively involved in ALS [9] including the fused in sarcoma (FUS) RNA-binding protein and the TAR DNA-binding protein 43 (TDP43). The earliest discovered genetic predisposition in ALS was found in Cu/Zn superoxide dismutase 1 (SOD1). Mutations in SOD1 make up approximately 20% of all fALS cases and are identified in 2–7% of sALS cases [10]. In 1994, Gurney et al. [11] created the first transgenic mouse model for ALS, the tg(SOD1*G93A)1Gur mice that develop ALS-like symptoms, by introducing a high copy number of the human mutated SOD1 gene (SOD1*G93A) into the mice’s genome. Through an accumulation of the mutated SOD1 protein in motor neurons, mice develop degeneration of UMNs and LMNs with successive activation of inflammation, neurofilament accumulation, paralysis of limbs, and, finally, a shortened life span. As the cause of sALS and the exact disease mechanisms prior to symptom onset are still unknown, mouse models are an important tool to study underlying disease mechanisms and to test potential drugs for ALS treatment.

At the moment, there are only three drugs available for the treatment of disease symptoms. Riluzole, an inhibitor of glutamate release at synapses [12], edaravone, a free-radical scavenger and antioxidant [13], and the latest, relyvrio, a combination of two existing drugs, phenylbutyrate and taurursodiol, that are suggested to hinder mitochondrial and endoplasmic reticulum (ER) stress [14]. These drugs’ modes of action tackle important disease mechanisms in ALS. First, the glutamate excitotoxicity, in which neurons fire excessively, thereby damaging signal transduction and promoting cell death [15]. Second, the influence of reactive oxygen species (ROS) which are characterized by oxidative stress triggering disease mechanisms such as mitochondrial dysfunction and necrosis [16]. Third, the structural and functional abnormalities of mitochondria and the ER. Other identified mechanisms enabling ALS progression are impaired axonal structure and transport [7] and, as earlier mentioned, the impact of inflammation. As ALS affects the central nervous system (CNS) in large parts, the glial cells (microglia, astrocytes) play an essential role in regulating inflammation. By releasing proinflammatory cytokines such as interleukin (IL)-6 and tumor necrosis factor alpha (TNF-α), and anti-inflammatory cytokines such as IL-10 and IL-13, they maintain the immune balance in the CNS. In ALS, it is known that glial cells and immune activation are early mechanisms interfering with disease progression. In the more progressed and end stages of ALS, chronic inflammation promotes, rather than slows down, disease progression, making the interference of immune responses an interesting target for new treatment strategies [6]. 

In previous studies, the d-peptide RD2RD2 was able to reduce the motoric phenotype of the SOD1*G93A mice assessed using motoric and behavioral tests, as well as reduce neurodegeneration and neuroinflammation determined using histochemical analysis. Additionally, it was able to delay disease onset [5]. The aim of the current study was (1) to prolong the treatment period to evaluate the effect of RD2RD2 on the survival of SOD1*G93A mice, and (2) to treat non-transgenic litter mates with RD2RD2 to verify the safety of RD2RD2. Altogether, this study was designed to shed light on whether RD2RD2 acts symptomatically or causal in the SOD1*G93A mouse model of ALS.

## 2. Materials and Methods

SOD1*G93A transgenic mice were treated perorally with RD2RD2 for 19 weeks. During this period, disease progression was analyzed in several behavioral and motoric tests and compared to non-transgenic littermates. For a detailed description of experimental procedures, we refer to our previous studies [4,5].

### 2.1. Ethical Approval 

All applicable international, national, and/or institutional guidelines for the care and use of animals were followed. All procedures performed in studies involving animals were in accordance with the ethical standards of the institution or practice at which the studies were conducted. All animal experiments, including details on design, protocols, and analysis plan, were performed in accordance with the ARRIVE guidelines, the German Law on the protection of animals (TierSchG §§ 7–9), and with prior approval from the local authority and ethics committee (Landesamt für Natur, Umwelt und Verbraucherschutz (LANUV), North Rhine-Westphalia, Germany; reference number: AZ 81-02.04.2020.A160 (approval 20 August 2020) and AZ 81-02.04.2019.A489 (approval 26 March 2020)).

The QPS Austria animal facility is fully accredited by the Association for Assessment and Accreditation of Laboratory Animal Care (AAALAC). The study was performed as non-GLP study. However internal procedures were based on Good Laboratory Practice (GLP) guidelines. This study was performed in accordance with the study plan and QPS Austria standard operating procedures (SOPs) in the currently valid versions. All procedures in this study comply with the Animal Care and Welfare Committee. Additional regulations and laws that applied for the reason of animal welfare were followed in accordance with the Austrian Animal Experiments Regulation: Verordnung des Bundesministers für Wissenschaft und Forschung zur Durchführung des Tierversuchsgesetzes 2012 ((Tierversuchs-Verordnung—TVV 2012), BGBl. II Nr. 522/2012 idF BGBl. II Nr. 15/2014 (VFB)), the Austrian Animal Experiments Law (Bundesgesetz über Versuche an lebenden Tieren (Tierversuchsgesetz 2012—TVG 2012) BGBl. I Nr. 114/2012) and the Austrian Animal Welfare Law on the protection of animals used for scientific purposes (Bundesgesetz über den Schutz der Tiere (Tierschutzgesetz—TSchG), BGBl. I Nr. 118/2004 Directive 2010/63/EU of the European Parliament and of the Council of 22 September 2010). Furthermore, the study was performed according to the regulations of the Austrian Genetic Engineering Law (BGBl. Nr. 510/1994).

### 2.2. Animals

Male mice of the congenic B6.Cg-Tg(SOD1*G93A)1Gur/J line were purchased from JAX (Strain #: 004435, The Jackson Laboratory, Bar Harbor, ME, USA) and bred in-house with C57BL/6J females obtained from CRIVER (Strain Code 632, Charles River Laboratories, Sulzfeld, Germany). Each mouse was genotyped using RT-PCR with DNA extracted from ear tissue collected at P21. The SOD1 copy number was checked prior to the study start to ensure a stable phenotype progression as previously described [17].

The mice were housed at the animal facility of the Forschungszentrum Jülich under a controlled specific-pathogen-free (SPF) environment (12/12 h light/dark cycle, humidity maintained around 50%, and a room temperature between 20 and 23 °C). Food, water, and cages were autoclaved prior to entering the SPF area. A maximum of five SOD1*G93A mice and non-transgenic littermates were housed in individually ventilated cages on standardized rodent bedding (Rettenmaier, Rosenberg, Germany). Dried, pelleted standard rodent chow (Altromin, Lage, Germany) as well as tap water were available for the animals ad libitum. After disease onset, nutrition of the animals was ensured using gel pads (Solid Drink® SDST-75, Triple A trading, Tiel, The Netherlands).

### 2.3. Drug Candidate

RD2RD2 was purchased from CBL Patras (Patras, Greece) as a lyophilized powder. The peptide consists of 24 d-enantiomeric amino acid residues with an amidated C-terminus (ptlhthnrrrrrptlhthnrrrrr, 3.2 kDa). The peptide solution was created by dissolving the powder in autoclaved bidest water. 

### 2.4. Study Design

For this study, 28 transgenic (tg) SOD1*G93A mice were divided into two groups of *n* = 14 each, placebo vs. RD2RD2. The non-transgenic (ntg) littermates were used in two groups of *n* = 7 each, whereby one group was treated with RD2RD2 to monitor potential adverse drug effects. Baseline tests prior to treatment start were conducted at the age of 7 weeks, and treatment started at the age of 8 weeks. Weekly tests (SHIRPA test, pole test, splay test, wire hanging test) during the treatment period were conducted from the age of 9 weeks to 20 weeks. Every four weeks, additional tests (open field test (OFT), rotarod) were included. Three times a week, the mice were weighed and checked for symptoms and general health conditions. Daily health checks were performed after disease onset to monitor termination criteria. Mice were euthanized when reaching humane endpoints (see below). The survival rate was determined for all tg SOD1*G93A animals. 

### 2.5. Treatment

Treatment was started at week 8, before symptom onset (defined after [18]). The ntg and tg animals were treated daily with either 50 mg/kg body weight RD2RD2 or placebo (bidest H_2_O). The peptide solution or placebo was added to gelatin drops which were fed to the mice individually in a clean cage (voluntary peroral administration). The drops contained 19% instant gelatin (Dr. Oetker, Bielefeld, Germany), 30% sucrose, and 10% sucralose. They were prepared in 96-well plates. After solidification, the drops were stored at 4 °C for a maximum of 6 days until feeding. 

### 2.6. SHIRPA Phenotype Assessment

With the help of the SmithKline Beecham, Harwell, Imperial College, Royal London Hospital, phenotype assessment (SHIRPA) test battery, several motoric and behavioral characteristics were evaluated. Every test was graded with a score (0 = ntg-like to 3 = extremely different from ntg animals) which was summed up for phenotype assessment to the SHIRPA score sum. Additionally, the motoric tests were evaluated separately to form the motor score sum. The SHIRPA test was performed weekly from baseline recording (week 7) to the end (week 20).

### 2.7. Modified Pole Test

In the modified pole test [19] the mice were placed with their head downwards onto a rough-surfaced pole. With a scoring system from 0 to 3 (0 = ntg-like, 1 = altered locomotion, 2 = sliding down the pole, 3 = falling down the pole), their locomotion and grip strength were analyzed. The test was repeated three times, and the score sum was built. The pole test was performed weekly from the age of 7 weeks to 20 weeks. 

### 2.8. Splay Reflex Test of Hind Limbs

The splay reflex is a susceptible test to monitor disease progression in the hind limbs. During disease progression tg animals lose their ability to stretch their hind limbs, i.e., they lose the splay reflex. This was evaluated with a score from 0 to 3 (0 = ntg-like, 1 = reduced splay, 2 = minimal splay, 3 = complete loss of splay). After three repetitions, the score sum was built. The splay reflex test was performed weekly from baseline (week 7) to the end of testing (week 20).

### 2.9. Wire Hanging Test 

For the wire hanging test [20], the mice were placed on a metal grid approximately 50 cm above the ground. After a short adjustment, the grid was turned upside down, and the mice had to hold their grip for 5 min. Cages containing rodent bedding were placed under the mice to prevent injuries when they fell. Because of its strenuous nature, this test was not repeated. The latency to fall was recorded. The test was performed every week from week 7 to week 20. 

### 2.10. Rotarod

The rotarod test was used to analyze the mobility of the mice by forced locomotion on an accelerating rod. In a period of 5 min, the latency to fall off the rod was recorded. The test was performed twice a day (morning and afternoon) with three repetitions each. The mean latency to fall was used to analyze locomotor function. The test was performed every fourth week starting at baseline recording until the mice’s disease symptoms were too severe (usually after 4 recordings).

### 2.11. Open Field Test

For the open field test (OFT), the mice were placed in a cubical arena and videotaped for later analysis. The arena was divided into a center and a border zone so that the mice’s behavior regarding their anxiety and exploration behavior could be analyzed. With EthoVision 15.0 (Wageningen, The Netherlands), the videos were analyzed and criteria such as exploration time and total distance moved could be generated. This test was repeated every fourth week, starting at baseline testing until the mice were too weak to participate (usually after 4 recordings). 

### 2.12. Disease Onset and Survival Analysis

Disease onset and survival analyses were performed using Kaplan–Meier analyses. Disease Onset was defined after [18] as the “point at which defects in hind limb splay and enhanced tremor were observed with a score of at least 1 in each category”. The day of disease onset (=score change from 0 to 1) was compared between the tg placebo and the tg RD2RD2 group. The study end-point for survival analyses was defined as the point when mice reached the defined end-point criteria (weight loss over 10% of body weight and/or partial paralysis of hind limbs for over 48 h, or loss of righting reflex) and had to be euthanized. 

### 2.13. External Study

To validate the survival data of the in-house study, an external study at QPS Austria GmbH (Grambach, Austria—member of the QPS Custom-Built ResearchTM, Newark, DE, USA) was commissioned to test RD2RD2 in a parallel study in tg SOD1*G93A mice on a mixed B6SJL genetic background and using both genders. B6SJL-Tg(SOD1*G93A)1Gur/J (Strain# 002726) mice purchased from Jackson Laboratory (Bar Harbor, ME, USA) at the age of 6 weeks were used. In total 32 tg animals (placebo *n* = 16, RD2RD2 treatment *n* = 16, 8 of each sex) and *n* = 8 ntg animals (4 of each sex) were utilized, whereby the tg animals were treated daily via oral gavage with 50 mg/kg body weight RD2RD2 or placebo (0.9% sodium chloride solution, pH 7.0–7.4). At the age of 9 weeks, the splay test was performed twice a week to assess disease onset. After 50% of animals reached onset criteria, the treatment with RD2RD2 started. From week 16 on, clinical signs were monitored, and mice with severe phenotypes (e.g., weight loss of more than 20%, limb paralysis, decrease in general behavior, motor/gait disturbances, hypothermia, dyspnea) were euthanized.

### 2.14. Statistics

The normal distribution of data was checked with InVivoStat v.4.4.0 (InVivoStat by Simon Bate and Robin Clark, Cambridge, UK [21]). All statistical analyses were performed with Sigma Plot v.11.0 (Systat Software, Erkrath, Germany). The weekly tests, body weight, OFT, and rotarod, were analyzed with a two-way repeated measure (RM) ANOVA and post-hoc Fisher’s least significant difference (LSD) test. Disease onset and survival were determined using Kaplan–Meyer analysis. For *p* ≤ 0.05, the results were considered significant. Data presented in graphs were visualized with GraphPad PRISM v.5.00 (GraphPad Software, Inc., La Jolla, CA, USA). 

## 3. Results

### 3.1. RD2RD2 Does Not Change Body Weight during Oral Administration

**Figure 1:**
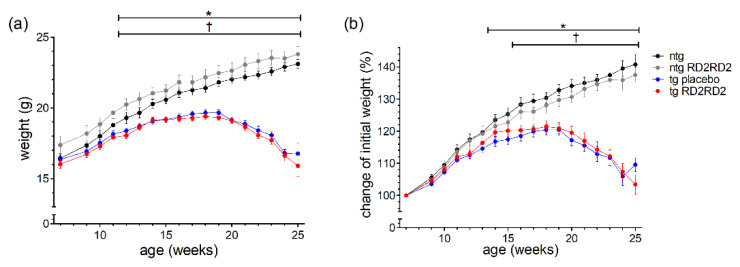
Body weight and the percentage of weight gain during oral treatment with RD2RD2: The mice were weighed three times a week. Data are presented as mean ± SEM. The total body weight (**a**) is compared to the percentage of weight gain of the initial body weight (**b**). Statistical analysis was performed using two-way RM ANOVA and Fisher’s LSD post-hoc test (n_ntg_ = 7, n_ntg_,_RD2RD2_ = 7, n_tg,placebo_ = 14, n_tg,RD2RD2_ = 13). Significant findings are displayed as asterisks (ntg vs. tg placebo, * *p* ≤ 0.05) and crosses (ntg vs. tg RD2RD2, ^†^ *p* ≤ 0.05). The differences between tg placebo- and tg RD2RD2-treated animals are non-significant. (**a**) F(48) = 31.540, *p* < 0.001; (**b**) F(48) = 31.540, *p* < 0.001.

The body weight of every animal was recorded three times a week. The mean weight per week is depicted in Figure 1 as total number (Figure 1a) and as percentage of weight change (Figure 1b). 

Only ntg animals showed a steady weight gain whereas tg animals lost weight rapidly from week 18 on. Considering the total body weight, the ntg RD2RD2 group showed an overall higher weight during the study period. This phenomenon cannot be seen when only looking at the percentage of weight gain. Treatment with RD2RD2 did not influence the body weight of either ntg or tg animals, so there was no significant difference between tg placebo-treated and tg RD2RD2-treated mice, and between ntg and ntg RD2RD2 mice. From week 12 on, both tg groups had a significantly lower mean total body weight in comparison to the ntg animals. This was also true for the percentage of weight change, whereas the comparison of ntg and tg placebo animals showed a significant difference from week 14 on, and the comparison of ntg and tg RD2RD2-treated animals from week 16 on.

### 3.2. RD2RD2 Reduces the Early Motor Phenotype in SOD1*G93A Mice

**Figure 2:**
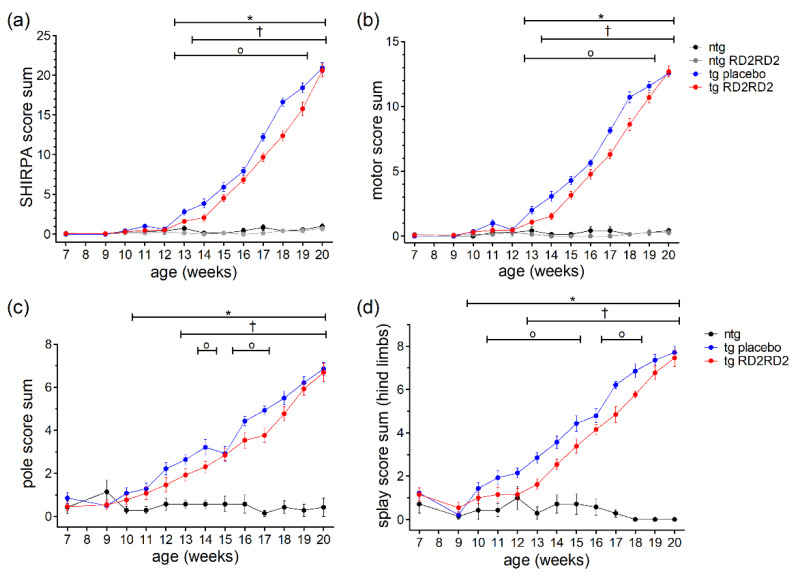
Motor phenotype in SOD1*G93A mice during oral treatment with RD2RD2: The mice’s phenotype was evaluated every week from the age of 9 weeks on with the SHIRPA test (**a**,**b**), the pole test (**c**), and the splay reflex test (**d**). Performance at the age of 7 weeks is considered baseline. Treatment started at week 8. Data are presented as mean ± SEM. Statistical analysis was performed using two-way RM ANOVA and Fisher’s LSD post-hoc test (n_ntg_ = 7, n_ntg,RD2RD2_ = 7, n_tg,placebo_ = 14, n_tg,RD2RD2_ = 13). In general, RD2RD2 (red) was able to reduce the disease phenotype in comparison to tg placebo (blue) animals (**a**–**d**). Significant findings are displayed as asterisks (ntg vs. tg placebo, * *p* ≤ 0.05), crosses (ntg vs. tg RD2RD2), and circles (tg placebo vs. tg RD2RD2, ^†^ *p* ≤ 0.05). (**a**) F(36) = 93.108, *p* < 0.001; (**b**) F(36) = 93.420, *p* < 0.001; (**c**) F(24) = 18.400, *p* < 0.001; (**d**) F(24) = 26.670, *p* < 0.001.

Phenotype assessment during disease progression was performed with the SHIRPA test (Figure 2a). From week 13 on, tg SOD1*G93A mice showed elevated SHIRPA score sums in comparison to ntg litter mates (* *p* = 0.002). One week later, the SHIRPA score sums became significant in the tg RD2RD2-treated mice compared to the RD2RD2-treated ntg mice (^†^ *p* = 0.004). 

When comparing tg placebo-treated mice with tg RD2RD2-treated mice, drug treatment significantly reduced the SHIRPA score sum for 7 weeks. This effect became more pronounced at later time points (weeks 17 to 19) than around disease onset (weeks 13 to 16). Assessment of the motor score by analyzing only the motoric tests from the SHIRPA test battery yielded similar results (Figure 2b). RD2RD2 treatment reduced the motor score sum in tg SOD1*G93A mice significantly from week 13 to week 19. Again, tg placebo-treated animals differed significantly from the ntg comparison group from week 13 on, and tg RD2RD2-treated mice from the placebo-treated ntg group from week 14 on. 

To analyze the motoric phenotype of the disease in SOD1*G93A mice in more detail, additional motoric analyses were performed including the pole test and the splay test (Figure 2c,d). In the pole test (Figure 2c) a significant difference between tg animals and ntg animals was detected from week 11 on. The tg RD2RD2 group differed significantly from week 13 on (^†^ *p* = 0.004) compared to the ntg animals. The test also detected some differences between the tg placebo group and the tg RD2RD2 group in week 14 (^o^ *p* = 0.019), week 16 (^o^ *p* = 0.021), and week 17 (^o^ *p* = 0.003). The splay test was even more sensitive. It detected elevated splay score sums in tg animals from week 10 on (* *p* = 0.033). The tg RD2RD2-treated mice showed elevated score sums only from week 13 on (^†^ *p* = 0.005). Treatment with RD2RD2 significantly reduced the splay score sum for 7 weeks.

### 3.3. RD2RD2 Does Not Enable Persistent Motor Activity

**Figure 3:**
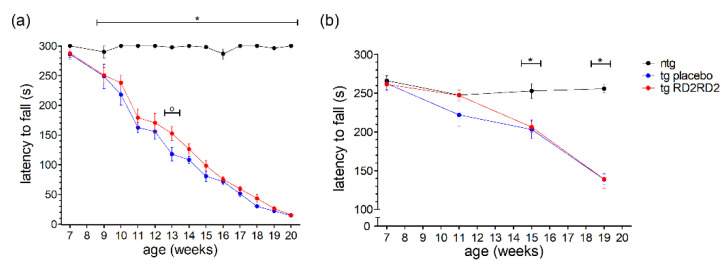
Motor activity of SOD1*G93A mice during oral treatment with RD2RD2: The wire hanging test was performed every week after treatment started at the age of 8 weeks (plus baseline at week 7) (**a**), the rotarod test was performed every fourth week (**b**). Data are presented as mean ± SEM. Statistical analysis was performed using two-way RM ANOVA and Fisher’s LSD post-hoc test (n_ntg_ = 7, n_tg,placebo_ = 14, n_tg,RD2RD2_ = 13). Significant findings are displayed as asterisks (ntg vs. tg placebo, * *p* ≤ 0.05) and circles (tg placebo vs. tg RD2RD2, ^o^ *p* ≤ 0.05). (**a**) F(24) = 23.316, *p* < 0.001; (**b**) F(6) = 12.320, *p* < 0.001.

In contrast to the earlier shown motoric tests such as the pole test and the splay reflex test (Figure 2), the wire hanging and rotarod tests analyze motoric features of the animals over a longer period (5 min). This ranks these tests at a higher severity level than the pole or SHIRPA test, meaning it is a strenuous task for the mice to perform. In the wire hanging test (Figure 3a), the tg animals showed a clearly reduced latency to fall in comparison to the ntg animals which had no problems in maintaining the grip on the grid for 5 min. 

This reduced latency to fall became more prominent the more the disease progressed. RD2RD2 did not have a strong beneficial effect; only at one time point a significant difference to the tg placebo-treated animals could be observed (week 13, ^o^ *p* = 0.007).

The rotarod test, even when executed only four times in the study period, displayed similar findings (Figure 3b). After the baseline recording, tg animals had reduced latencies to fall in comparison to the ntg animals. Overall, treatment with RD2RD2 did not change rotarod performance in SOD1*G93A mice. 

### 3.4. RD2RD2 Delays Disease Onset but Does Not Prolong Survival

**Figure 4:**
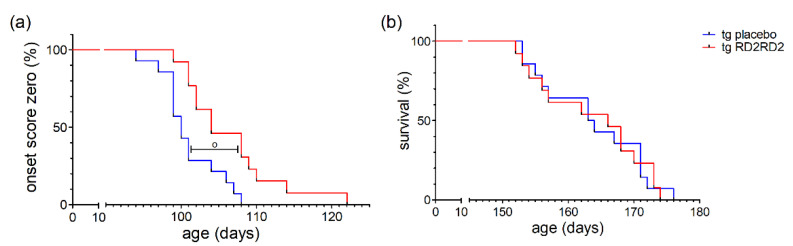
Disease onset and survival in SOD1*G93A mice after oral treatment with RD2RD2: Disease onset is delayed by RD2RD2 treatment (^o^ *p* = 0.005) (**a**). In the end stage of the disease, RD2RD2 is not able to prolong survival (*p* = 0.948) (**b**). Statistical analysis was performed using Kaplan–Meier analysis and log-rank test (n_tg,placebo_ = 14, n_tg,RD2RD2_ = 13).

Disease onset was defined as the first occurrence of splay deficit in one or two hind limbs in combination with tremor [18]. Treatment with RD2RD2 significantly delayed disease onset in the SOD1*G93A mice (^o^ *p* = 0.005) (Figure 4a). This was also clearly visible in the mean age of onset in the tg placebo group (101.0 days) versus the tg RD2RD2 group (106.5 days).

Comparison of the mean survival showed no significant difference between the RD2RD2-treated (163.5 days) and the placebo-treated (163.7 days) tg mice. This is also visible in the Kaplan-Meier survival analysis (Figure 4b) lacking any difference (*p* = 0.948) between tg treatment groups. Treatment with RD2RD2 was not able to prolong the survival of SOD1*G93A mice. 

### 3.5. RD2RD2 Does Not Change WT-like Behavior of the Mice

**Figure 5:**
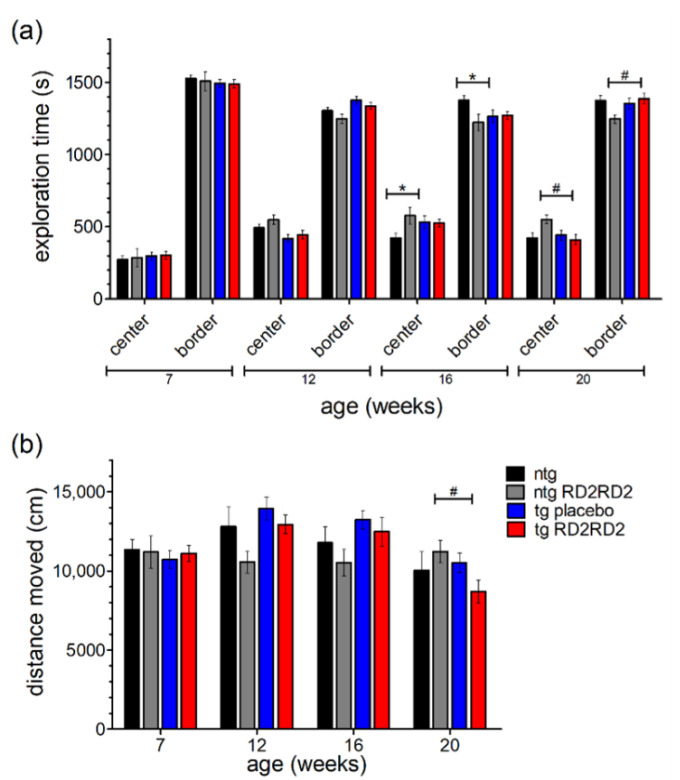
Behavior of SOD1*G93A mice in the open field test (OFT) during oral treatment with RD2RD2: OFT was repeated every fourth week. The analysis of exploration time is divided into a center and a border zone (**a**). The total distance moved is displayed in (**b**). Data are presented as mean ± SEM. Statistical analysis was performed using two-way RM ANOVA and Fisher’s LSD post-hoc test (n_ntg_ = 7, n_ntg,RD2RD2_ = 7, n_tg,placebo_ = 14, n_tg,RD2RD2_ = 13). Significant findings are displayed as asterisks (ntg vs. tg placebo, * *p* ≤ 0.05) and lozenges (ntg RD2RD2 vs. tg RD2RD2, ^#^ *p* ≤ 0.05). There are no significant differences between the tg placebo group and the tg RD2RD2 group. (**a**) F(9) = 2.206, *p* ≤ 0.05; (**b**) F(9) = 3.520, *p* < 0.001.

With the help of the open field test, the mice’s behavior in regard to anxiety and exploration can be analyzed. A higher exploration time in the center region points out a reduced anxiety behavior of the mice. Here, other than a small habituation effect in all groups seen by a slightly increased time in the center over time (Figure 5a), no significant differences in anxiety-related behavior were observed between groups. Only singular significant differences at week 16 and week 20 could be observed (ntg vs. tg placebo * *p* = 0.035; ntg RD2RD2 vs. tg RD2RD2 ^#^ *p* = 0.012) from which no general behavioral change can be concluded.

The total distance moved (Figure 5b) was similar in all treatment groups at baseline testing in week 7. Minor changes occurred in later trials but only at week 20 the tg RD2RD2 mice moved a significantly shorter distance (^#^ *p* = 0.029) than the ntg RD2RD2 mice.

### 3.6. No Rescue of Survival Time by RD2RD2 in SOD1*G93A/B6SJL Mice

**Figure 6:**
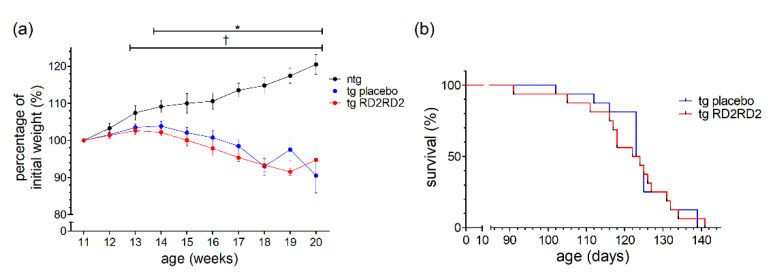
Evaluation of weight and survival of SOD1*G93A/B6SJL mice when treated with RD2RD2 from disease onset on: Only ntg animals gained weight (**a**). There was no change in survival rate (**b**). Data are presented as mean ± SEM. Statistical analysis for weight data was performed using two-way RM ANOVA and Fisher’s LSD post-hoc test (n_ntg_ = 8, n_tg,placebo_ = 16, n_tg,RD2RD2_ = 16). Survival analysis was performed using Kaplan–Meier analysis and log-rank test (n_tg,placebo_ = 16, n_tg,RD2RD2_ = 16). Significant findings are displayed as asterisks (ntg vs. tg placebo, * *p* ≤ 0.05) and crosses (ntg vs. tg RD2RD2, ^†^ *p* ≤ 0.05). (**a**) F(18) = 20,668, *p* < 0.001; (**b**) *p* = 0.824.

In the conformational study, RD2RD2 treatment started in female and male SOD1*G93A mice on a mixed B6SJL genetic background at the age of 11 weeks, after 50% of the animals showed disease onset. From disease onset on, the percentage of weight change was only increasing for ntg animals (Figure 6a). 

Both tg groups (placebo- and RD2RD2-treated) lost weight from the age of 14 weeks on, which was also visible in the significant differences starting at week 14 (* *p* = 0.019) when comparing ntg with tg placebo animals. When analyzing the survival of the tg animals (Figure 6b), treatment with RD2RD2 could not prolong survival. The mean age of survival of the tg RD2RD2 group (121.1 days) was not statistically different from the tg placebo group (124.1 days).

## 4. Discussion

Amyotrophic lateral sclerosis (ALS) is a fatal and progressive neurodegenerative disease affecting the motoric system of patients. In most ALS cases, the cause is unknown and diagnosis only possible after symptom onset [8]. Despite latest advances due to the approval of the drug relyvrio [22], the treatment of ALS remains symptomatic. The ongoing research on ALS disease mechanisms is of high importance for discovering new therapies to enhance a patient’s quality of life and to prolong life extensively in the future. All-d-peptides are an experimental drug class that gained interest in the last few decades. So far, the most promising all-d-peptide drug is RD2, which successfully passed phase I clinical trials in healthy human volunteers [3], and was developed as a treatment against AD. The head-to-tail tandem version RD2RD2 also showed promising results in an oral treatment study in an ALS mouse model [5]. In this study, female SOD1*G93A mice were treated with RD2RD2 for 10 weeks only, and in addition to behavioral and motor coordination tests performed regularly, the brains and spinal cords were histologically examined for gliosis and neurodegeneration at the end of the study to examine the anti-inflammatory effect of RD2RD2.

In the current studies, we prolonged the treatment period with RD2RD2 to assess its effect on survival in female SOD1*G93A mice and female and male tg SOD1*G93A mice on a mixed B6SJL genetic background. In addition, this is the first time RD2RD2 was administered to ntg animals to monitor their behavior and exclude adverse drug reactions. 

The time course of disease progression in the SOD1*G93A mouse model was extensively studied [17,23,24]. Loss of innervation at neuromuscular junctions and muscle atrophy, especially in hind limbs, are two of the earliest disease symptoms detectable from the age of 60 days [18]. According to these criteria, Mead et al. [18] defined disease onset as the time point at which the splay deficit in the hind limbs was visible with simultaneous occurrence of tremors. This is usually the case for the congenic B6.Cg-Tg(SOD1*G93A)1Gur/J line around the age of 100 days, whereby male mice show an earlier onset than female mice [25]. Treatment start was chosen to be at week 7, defined to be in the pre-symptomatic stage of the SOD1*G93A mice [26]. Towards the end of disease progression, the mice develop excessive weight loss, paralysis of limbs, and malnutrition, among other things [11]. Mice were sacrificed after pre-defined termination criteria before end-stage symptoms set in.

Treatment with RD2RD2 did not change the body weight of the mice. RD2RD2-treated ntg mice compared to placebo-treated ntg mice, and RD2RD2-treated tg mice in comparison to placebo-treated tg mice did not show significant differences in total body weight (Figure 1a). The significantly lesser weight of SOD1*G93A mice in contrast to ntg mice from week 12 on is a well-characterized feature of this mouse model [24,27]. Interestingly, there seems to be a tendency of RD2RD2-treated tg animals to have a slightly enhanced body weight in comparison to placebo-treated tg animals when looking at the percentage of weight change (Figure 1b). This is also seen in the start of statistical significance in week 16 for the percentage of weight change of the tg RD2RD2 group, in contrast to the tg placebo group on week 14 when compared to the ntg control group. A subsequent *t*-test of the mean body weights of the tg placebo group and the tg RD2RD2 group from week 13 to week 23 showed a significant difference of *p* = 0.047. This may indicate a minor effect of RD2RD2 treatment, resulting in a potential reduction in muscle wasting or an additional uptake of calories due to RD2RD2. 

Daily oral treatment with RD2RD2 from the age of week 6 resulted in a significant mitigation of the disease phenotype in the pre-symptomatic stage, during disease onset, and in early symptomatic stages as shown by the SHIRPA test, the pole test, and the splay test (Figure 2). Around weeks 19 to 20, this beneficial effect seems to get lost as the difference between the tg RD2RD2-treated mice and the tg placebo-treated mice becomes smaller. This early intervention window of RD2RD2 suggests modulation of early disease mechanisms in ALS. Previously, we could show the anti-inflammatory effect of RD2RD2 through a reduction in proinflammatory markers in plasma, and through the rescue of neurons in the cortex and brain stem [5]. RD2RD2 seems to be able to reduce chronic inflammation and thereby reduce neuronal loss in the early phases of the disease which is consequently slowing down disease progression in the first four months. This slowed-down progression is underlined by the delayed disease onset in SOD1*G93A mice when treated with RD2RD2 compared to the placebo group (Figure 4a). At later stages, the beneficial effect of RD2RD2 is obviously not sufficient to halt the disease progression in the SOD1*G93A mice. Disease progression in RD2RD2-treated mice caught up with the progression in placebo-treated tg mice. In the end stage of the disease, RD2RD2 was not able to prolong the survival of the mice (Figure 4b). As ALS is a multifactorial disease [28] it seems to not be sufficient to tackle only one underlying mechanism. Other mechanisms might overpower the one that is inhibited, thereby losing the beneficial effect of the drug. This is also seen in treatment with edavarone or riluzole, which mainly slow down disease progression or prolong survival mildly by 2–3 months, respectively [29]. The combinational drug relyvrio, on the other hand, delays death significantly by 6.5 months [14] showing a potential advantage of treating several disease mechanisms at once with co-medications.

No effect of RD2RD2 could be seen in the analysis of the persistent motor activity in the wire hanging test and the rotarod test (Figure 3). The latency to fall did not differ between the tg RD2RD2 and the tg placebo group. This hints at RD2RD2 having no beneficial effect on strenuous muscle tasks over a longer time period. However, in the short-timed motor tasks of the SHIRPA test and pole test significant improvement with RD2RD2 treatment was detectable (Figure 2). RD2RD2 seems to bring a benefit in instantaneous muscle strength rather than in unremitting muscle endurance pointing to a hindered effect of RD2RD2 under anaerobic muscle conditions. This might also exclude an effect of RD2RD2 on the mitochondria and their oxidative metabolism which are known to be implicated in ALS [30].

This was the first time that RD2RD2 was administered to ntg mice. In the phenotype assessment of the SHIRPA test (Figure 2) no abnormalities were detected. Ntg mice treated with RD2RD2 showed similar score sums as the untreated ntg mice, thereby showing no adverse drug effects in the phenotype assessment. The same was true for the behavioral assessment of the OFT test (Figure 5). There were no differences in center and border exploration, and in the total distance moved between the ntg-treated mice and the ntg-untreated mice. This suggests that RD2RD2 does not influence the mice’s anxiety, apathy, or alertness behavior. A comparable effect was visible for the tg mice. RD2RD2 did not influence explorative behavior in treated tg mice in comparison to the untreated mice. The only significant differences detectable in the OFT test exist between ntg and tg groups which hints at a decreased exploration of tg mice in later stages of disease progression due to motoric deficits in the limbs. Overall, we could not detect any adverse drug reactions of RD2RD2 in ntg and tg mice in any of the performed tests. 

Compared to our internal study, the confirmatory external study was conducted with five major changes. First, both genders were used, male and female SOD1*G93A mice. Second, SOD1*G93A mice on a mixed B6SJL genetic background were used instead of the congenic B6.Cg-Tg(SOD1*G93A)1Gur/J line. Third, disease onset was defined solely after the splay deficit without taking the hindlimb tremor into account. Fourth, the application of RD2RD2 solution was performed by forced swallowing through oral gavage. Fifth, RD2RD2 treatment started when 50% of the tg animals showed disease onset criteria. In spite of these variations, the body weight of tg placebo animals and tg treated animals was significantly lower in comparison to the ntg mice (Figure 6a). This is in accordance with the first study as well as with the characteristics of this mouse model [24]. RD2RD2 had no impact on body weight. Further, the external study did not show prolonged survival through RD2RD2 treatment in the SOD1*G93A mice (Figure 6b) which validates the results observed in the SOD1*G93A B6 mice.

So far, the exact target for RD2RD2 has not been discovered yet. Further research into this direction is needed in the future to unravel the exact mechanisms by which the d-peptide reduces early motor symptoms and delays disease onset. On this basis, the drug might be improved to enhance and prolong the therapeutic effect.

## 5. Conclusions

RD2RD2 demonstrates some potential to decelerate the worsening of some disease symptoms. This effect is strong enough to delay disease onset in SOD1*G93A mice. It does not lead to permanent differences in placebo-treated mice and does not prolong survival of the mice. There are no adverse reactions considering the mice’s behavior in ntg and tg animals. A pre-symptomatic start of treatment with RD2RD2 might be crucial for an improved effect of the drug. Future advances in prognostic and diagnostic markers would be beneficial for an early treatment start with RD2RD2. Additionally, RD2RD2 might be optimized to increase the efficacy concerning reduced inflammation and rescued motor activity.

## Data Availability

This study includes no data deposited in external repositories. The datasets generated and/or analyzed during the current study are available from the corresponding author on reasonable request.
